# Factors influencing uptake of intermittent preventive treatment of malaria in pregnancy using sulphadoxine pyrimethamine in Sunyani Municipality, Ghana

**DOI:** 10.11604/pamj.2017.28.122.12611

**Published:** 2017-10-10

**Authors:** Hajira Ibrahim, Ernest Tei Maya, Kofi Issah, Paschal Awingura Apanga, Emmanuel George Bachan, Charles Lwanga Noora

**Affiliations:** 1 Regional Health Directorate, Upper East Region, Bolgatanga, Ghana; 2 University of Ghana, Department of Population, Family and Reproductive Health, Accra, Ghana; 3 Talensi District Hospital, Tongo, Ghana; 4 Regional Health Directorate, Brong Ahafo Region, Sunyani, Ghana; 5 Sunyani Municipal Hospital, Sunyani, Ghana

**Keywords:** Sulphadoxine pyrimethamine, intermittent preventive treatment, malaria, pregnancy, uptake

## Abstract

**Introduction:**

Malaria continues to pose a public health challenge in Ghana particularly in pregnant women. Ghana adopted intermittent preventive treatment of malaria in pregnancy policy using sulphadoxine pyrimethamine. Despite its implementation, its coverage still remains low. This study sought to investigate factors that influence the uptake of intermittent preventive treatment of malaria in pregnancy in the Sunyani Municipality.

**Methods:**

This was a cross sectional study which employed a quantitative method. The study was conducted in five selected facilities in the Sunyani Municipality within the period of January to June 2015. Structured questionnaires were administered to 400 pregnant women randomly sampled from antenatal clinics of selected health facilities. Descriptive, bivariate and multivariate analysis of quantitative data was done using Stata12.

**Results:**

A total of 400 pregnant women at 36 weeks or more gestational age were studied. The study revealed that 98.5% of the pregnant women received at least one (1) dose of sulphadoxine pyrimethamine during the current pregnancy with 71% receiving optimal (at least 3 doses) doses of sulphadoxine pyrimethamine for intermittent preventive treatment of malaria in pregnancy at the time of study. The study revealed that women who attended ANC ≥4 times (Adjusted OR = 4.7, 95% CI 1.31-17.2), knowledge of malaria in pregnancy (Adjusted OR = 2.2, 95% CI 1.03-4.62) and knowledge of intermittent preventive treatment for malaria in pregnancy (Adjusted OR = 1.8, 95% CI 1.15-2.96) were found to be positively associated with the uptake of optimal doses of sulphadoxine pyrimethamine.

**Conclusion:**

This study has demonstrated that having a good knowledge of malaria in pregnancy and intermittent preventive treatment of malaria in pregnancy can significantly influence the uptake of optimal doses of sulphadoxine pyrimethamine. Encouraging women to attend antenatal care regularly (at least four visits) could also increase the optimal uptake of sulphadoxine pyrimethamine.

## Introduction

Malaria is an enormous international health problem affecting mainly young children, pregnant women and adults with little or no immunity. Over 25 million pregnant women are at risk of the infection each year worldwide. Malaria in pregnancy may result in maternal and foetal anaemia, prematurity, stillbirths, rarely congenital malaria as well low birth weight which is the single greatest risk factor for neonatal deaths [1, 2]. More than 30 million women in Africa become pregnant in malaria endemic areas and are at risk of Plasmodium falciparum malaria infection compared to non-pregnant women. Yet only a fraction of these women have access to effective anti-malaria interventions [3]. The susceptibility of pregnant women to Plasmodium Falciparum malaria increases the risk of disease and a high incidence of death for both the mother and her foetus [4]. In Ghana, malaria among pregnant women accounts for about 14% of Out Patient Department (OPD) attendance, 11% of admissions and 9% of deaths [5]. Malaria in pregnancy may occur depending on a woman's exposure to mosquitoes, her level of immunity and possible co-infections such as other malaria species, HIV or helminths as well as the efficacy of treatment and preventive interventions available to her [6]. Even though adult women in malaria endemic areas have a high level of immunity, this generally is impaired during pregnancy particularly in the first pregnancy, thereby increasing their risk of infection [7]. This increased risk has been attributed to the immunological, hormonal and physiological changes in pregnancy [8]. The reduced immunity may result in the risk of acute and severe clinical disease as well as more frequent episodes [2]. Although malaria in pregnancy may sometimes be asymptomatic, it can still affect the health of a woman and that of her unborn child. Following the recommendation of the World Health Organization (WHO) in 2000, Ghana adopted a new malaria treatment policy in 2004. The country thus moved from the use of mono-therapy to combination therapy using Artemisinine-Based Combination Therapy [1]. As part of this policy was the change from use of weekly Chloroquine Chemoprophylaxis to Sulphadoxine-Pyrimethamine (SP) as Intermittent Preventive Treatment (IPT) for malaria prevention during pregnancy.

This was based on growing concerns of resistance to Chloroquine and the fact that only 11.6% were adhering to the policy of using Chloroquine as IPT [1]. The new policy of Intermittent Preventive Treatment in pregnancy (IPTp) using SP was piloted in 20 selected districts of Ghana and by 2005 scaled up to a nationwide coverage. A revision of the policy was done in 2007 and the revised current policy was formulated in 2012 [9]. According to the policy, IPT was to be implemented as part of a multipronged approach as recommended by WHO to reduce the burden of malaria in pregnancy. These approaches consist of three tenets; which include the use of SP as IPTp, Insecticide Treated Nets (ITN) and case management of malaria illness. A clinic-based prevention approach was adopted as over 90% of the target (pregnant women) attend antenatal clinic at least once during pregnancy [1, 10]. Intermittent preventive treatment of malaria in pregnancy is based on the assumption that every pregnant woman living in areas of high malaria transmission has malaria parasites in her blood or placenta, whether or not she has symptoms of malaria. It involves the administration of treatment doses of SP in at least monthly intervals during pregnancy with the first dose administered as early as possible in the second trimester and the last dose administered up to the time of delivery [11]. The potential of IPT to achieve great levels of programme coverage due to high level of acceptability by women and its benefits in reducing malaria in pregnancy which causes maternal anaemia and Low Birth Weight (LBW) makes it a preferred strategy [5]. According to the Ministry of Health, every pregnant woman is expected to receive at least two doses of SP under Directly Observed Therapy (DOT) [1]. Although Ghana has adopted WHO recommendations on the use of SP for IPTp, IPT2 coverage remains generally low [12]. Like many other endemic countries, Ghana's ability to achieve the global target of 80% IPT2 coverage still poses a great challenge [13-15]. However, not much has been documented about the knowledge of pregnant women on IPT and malaria in pregnancy as well as factors that influence the uptake of IPT of Malaria in Pregnancy using SP among pregnant women. This study therefore will determine the factors that influence the uptake of IPT of Malaria in Pregnancy using SP among pregnant women.

## Methods

*Settings, population and study design*: this was a cross-sectional study that was conducted in the Sunyani Municipality between January and June, 2015. Questionnaires were administered to pregnant women of 36 weeks gestation and above who reported to antennal clinics of some selected health facilities in the Sunyani Municipality. The Sunyani Municipality is the capital of the Brong Ahafo region of Ghana. It is bordered to the North by Sunyani West District, Asutifi District to the South, Tano North District to the East and Dormaa East District to the West. The population of the municipality is 131,924 with a total of 32,716 being Women in the Fertile Age (WIFA), with an expected number of 4,089 pregnancies annually [16].

*Sample size*: a sample size of 384 was calculated based on the sample size formula for a single population. This was estimated using an expected IPT3 prevalence of 50% in the Sunyani Municipality [17]. This sample had a margin of error of 5% and confidence interval of 95%. This was calculated using the formula:

$$n=\frac{z^{2}p(1-p)}{E^{2}}$$

Where: n = the minimum required sample size. z = standard normal deviation corresponding to 95% confidence interval, which equals to 1.96. P= proportion of pregnant women who received IPT3 taken to be 50% E = the margin of error on P estimated to be at 5%.

Therefore:

$$n=\frac{1.96^{2}(0.5)(1-0.5)}{{\left(0.05\right)}^{2}}$$

n = 384. Adjusting for 10% of non-response rate, a total 422 participants were required for the study.

*Sampling method*: a total of 400 participants were recruited for the study. Five health facilities were randomly sampled from health facilities with antennal clinics from the Sunyani Municipality. Health facilities that were selected include; Brong Ahafo Regional Hospital, Sunyani Municipal hospital, SDA hospital, Owusu Memorial Hospital and Monica Maternity home. Study participants were randomly sampled from antenatal clinics of these health facilities. The inclusion criterion for sampling was pregnant women who had 36 weeks gestation and above were selected for study. The first pregnant woman who was met at the antenatal clinic was recruited for the study provided she met the inclusion criterion and consented for the study.

*Measurement*: pre-coded structured questionnaires were administered to participants by the researchers. The questionnaires were divided into two main parts. The first part was on socio-demographics characteristics of respondents including the coverage of SP whilst the second part was on knowledge of Malaria in pregnancy (Mip) and IPTp. The questionnaires were administered to participants in a language that they could comprehend easily. The questionnaires were pre-tested in the Sunyani West District, a neighbouring district to enable the research team identify problems and refine the questionnaire.

*Data analysis*: the data collected was cleaned and analyzed by STATA 12. Descriptive statistics was used to summarize and present data on socio-demographic characteristics of respondents as well as their knowledge on Mip and IPTp. The association between the binary outcome {sub optimal (≤2 doses) or optimal (≥3 doses)} and independent variables (age, parity, marital status, educational status, employment status, gestational age at 1^st^ANC visit, number of ANC visits, knowledge of Mip and knowledge of IPTp) were assessed using the chi-square test. Bivariate and multivariate logistic regression were used to determine the factors that influenced the uptake of SP. The P-value of 0.05 was taken for statistical significance.

*Ethics*: ethical clearance was obtained from the Ghana Health Service Ethical Review Committee. Permission was also obtained from the Regional Director of Health Services of the Brong Ahafo Region as well as the Sunyani Municipal Director of Health Services. Permission was also sought from health facility managers where the study was conducted. Written informed consent was obtained from study participants after they were well informed about the study. Participants were not pressured to take part in the study and were informed about the purpose of the study. They were also assured of full confidentiality of information they provided and their right to withdraw from the study even after they have participated.

*Limitations of the study*: some of the pregnant women might not have given accurate responses because they might be aware of the national policy on SP in pregnancy or they might be afraid that their responses might incriminate their healthcare providers. However, we assured them of privacy and confidentiality of the information they provided. Some of the study participants did not understand the English language so we had to make use of the local language to interview them.

## Results

**Figure 1:**
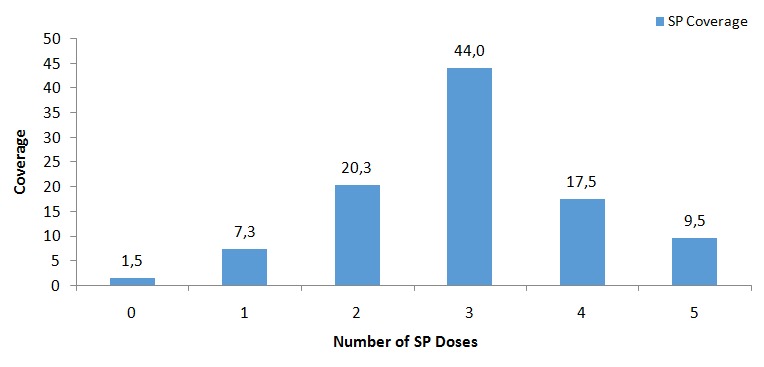
SP coverage

Table 1 shows socio-demographic characteristics of 400 participants who took part in the study. Most of the respondents were within the age group of 25-29years, 37.8% (151/400). Of the 400 participants who took part in the study, 86.5% (346/400) of them were married whilst 9% (36/400) were single. Most of the participants were multiparous, 45.2% (181/400). Most of the participants involved in the study were educated at postbasic level, 56.5% (226/400). Majority of the participants were employed, 90.3% (361/400). Majority of the respondents were within the age groups of 37.8% (151/400) and 30.3% (121/400). The mean age of respondents in the study was 29.1 ± 4.9 with a range of 16-44 years. Table 2 summarizes responses of participants' knowledge on malaria in pregnancy and the use of SP for intermittent preventive treatment of malaria among pregnant women. With regards to knowledge on malaria in pregnancy, 89% (365/400) of the participants said malaria was transmitted through a mosquito bite. Majority of the participants, 55% (220/400) attributed anaemia in pregnant women to be one of the adverse effects of malaria in pregnancy whilst, 33.8% (135/400) of the participants opined that malaria can cause spontaneous abortion in a pregnant woman. Most of the participants prefer sleeping under insecticide treated nets as a means of preventing malaria in pregnancy. On the part of knowledge of participants on IPTp, 76% (304/400) knew the purpose of IPTp and 39% (196/400) said such treatment starts at 16weeks or greater. Many participants, 95.3% (381/400) were aware of the one monthly interval to receive SP after starting treatment, however, few participants knew the number of required doses of SP, 35.5% (142/400) in Ghana. Table 3 shows the Bivariate model. This model provides results of the association between client characteristics and the binary outcome {sub optimal (≤ 2 doses) or optimal (≥ 3 doses)} using chi-square test. It showed that educational status, antenatal care attendance of 4 or more visits, having good knowledge of MiP and IPTp were positively associated with optimal uptake of SP (P < 0.05). However, age, parity, marital status, employment status and gestational age at 1st visit of study participants were not statistically significant. Table 4 shows the multivariate model. This summarizes results of a further logistic regression of client characterizes and its influence on the uptake of IPTp using SP. It showed that antenatal care attendance of 4 or more visits, having good knowledge of MiP and IPTp were positively associated with optimal uptake of SP (P < 0.05). However, age, parity, marital status, educational status, employment status and gestational age at 1^st^ visit were not statistically significant. The Coverage of SP is summarized in Figure 1 below. The uptake of SP by the pregnant women ranged between 0 and 5 doses. An average of 3 doses received is considered optimal IPT uptake. Majority of the women, 44% (176/400) had received 3 doses while only 1.5% (6/400) received no dose of SP at 36 weeks gestation or above.

**Table 1:** Socio-demographic characteristics of study participants, Sunyani

Characteristic	Frequency	%
**Age category**		
15-19	6	1.5
20-24	67	16.8
25-29	151	37.8
30-34	121	30.3
35+	55	13.8
**Parity**		
Nullipara	99	24.8
Primapara	120	30.0
Multipara	181	45.2
**Marital Status**		
Married	346	86.5
Single	36	9.0
Co-habiting	12	3.0
Widowed	6	1.5
**Educational status**		
None	35	8.8
Basic	139	34.7
Post Basic	226	56.5
**Employment status**		
Employed	361	90.3
Unemployed	39	9.7

NB: Basic = primary and junior high schools, postbasic = senior high and tertiary schools

**Table 2:** Knowledge of Mip and IPTp

Knowledge Variable	Responses	N	%
**Malaria in Pregnancy**			
Cause of malaria	Through bite of a mosquito	356	89.0
Effects of malaria on pregnant woman	Can cause anaemia	220	55.0
	Preterm labour	70	17.5
	Can cause death	64	16.0
Effects of malaria on unborn baby	Can cause spontaneous abortion	135	33.8
	Can cause intra uterine death	38	9.5
	Can cause low birth weight	60	15.0
	Can cause prematurity	98	24.5
Malaria Preventive methods	Sleep under an ITN	348	87.0
	Use mosquito repellent	35	8.8
	Wear protective clothing	8	2.0
**IPTp**			
Purpose of IPT	To prevent mother and baby from malaria	304	76.0
Time to start	≥16 weeks	196	49.0
Number of Doses in Ghana	5 doses	142	35.5
Interval between doses	1 month	381	95.3

MiP= Malaria in Pregnancy, IPTp= Intermittent Preventive Treatment in pregnancy, ITN= Insecticide Treated Net NB: Some respondents gave more than one reason

**Table 3:** Bivariate analysis of factors that influence the uptake of SP

	SP Doses		
	Sub Optimal (≤2 doses)	Optimal(≥3 doses)	P value
**Age**			0.20
<30 years	56(25.0)	168(75.0)	
≥30 years	54(30.7)	122(69.3)	
**Parity**			0.21
≤1	55(25.1)	164(74.9)	
≥2	62(34.3)	119(65.7)	
**Marital status**			0.37
Married	96(27.7)	250(72.3)	
Not married	15(27.8)	39(72.2)	
**Educational Status**			0.01
Educated	95(26.1)	270(73.9)	
Not Educated	16(45.7)	19(54.3)	
**Employment status**			0.15
Employed	104(28.8)	257(71.2)	
Unemployed	9(23.1)	30(76.9)	
**Gestational Age at 1st ANC visit**			0.06
Early (1^st^&2nd trim)	103(27.1)	277(72.9)	
Late (3rd trim)	10(50.0)	10(50.0)	
**Number of ANC Visits**			<0.01
≥ 4Visits	102(26.7)	280(73.3)	
<4 visits	14(77.8)	4(22.2)	
**Knowledge of IPTp**			0.01
Good (>50%)	52(23.2)	172(76.8)	
Poor (≤50%)	64(36.4)	112(63.6)	
**Knowledge of MiP**			0.03
Good (>50%)	95(26.4)	265(73.6)	
Poor (≤50%)	15(37.5)	25(62.5)	

**SP=** Sulphadoxine-Pyrimethamine, ANC=antenatal care

**Table 4:** Multivariate analysis of factors that influence the uptake of IPTp using SP

	SP Doses		Unadjusted Odd ratio		Adjusted Odd ratio	
Variable	Sub Optimal(≤2 doses)	Optimal(≥3 doses)	(95% CI)	P- value	(95%CI)	P value
**Age**						
≥30 years	54(30.7)	122(69.3)				
<30 years	56(25.0)	168(75.0)	0.75(0.48-1.17)	0.20	0.77(0.46-1.28)	0.32
**Parity**						
≥2	62(34.3)	119(65.7)				
≤1	55(25.1)	164(74.9)	0.75(0.48-1.17)	0.21	0.91(0.54-1.54)	0.73
**Marital status**						
Not married	15(27.8)	39(72.2)				
Married	96(27.7)	250(72.3)	1.42(0.66-3.09)	0.37	0.95(0.42-2.12	0.43
**Educational Status**						
Not Educated	16(45.7)	19(54.3)				
Educated	95(26.1)	270(73.9)	0.42 (0.21-0.86)	0.01	0.5(0.25-1.13)	0.10
**Employment status**						
Unemployed	9(23.1)	30(76.9)				
Employed	104(28.8)	257(71.2)	0.7(0.40-1.15)	0.22	0.63(0.37-3.52	0.11
**Gestational Age at 1st ANC visit**						
Late (3rd trim)	10(50.0)	10(50.0)				
Early (1^st^&2nd trim)	103(27.1)	277(72.9)	0.40(0.15-1.22)	0.10	0.44(0.14-1.47)	0.19
Number of ANC Visits						
<4 visits	14(77.8)	4(22.2)				
≥ 4Visits	102(26.7)	280(73.3)	5.5(1.6-18.8)	<0.01	4.7(1.31-17.2)	0.01
**Knowledge of IPTp**						
Poor (≤50)	64(36.4)	112(63.6)				
Good(>50)	52(23.2)	172(76.8)	2.03(1.30-3.19)	<0.01	1.8(1.15-2.96)	0.01
**Knowledge of MIP**						
Poor (≤50)	15(37.5)	25(62.5)				
Good(>50)	95(26.4)	265(73.6)	2.19(1.06-4.5)	0.02	2.2(1.03-4.62)	0.04

## Discussion

Findings from this study suggest that 98.5% of pregnant women received at least one dose of SP in the Sunyani Municipality. Approximately 91% of pregnant women received at least 2 doses of SP and 71% received 3 or more doses of SP. The national target of IPTp is 100% of at least two dose of SP. The coverage of 91% of at least IPT2 suggests that although IPTp-SP usage is high, it is still below the national target [17]. Also, the coverage of 71% receiving at least 3 doses of SP in this study is higher than the national figure (39%) reported for IPTp 3 coverage in the 2014 Ghana Demographic and Health Survey and also higher than 23% of IPTp 3 reported by WHO [11, 18]. The difference in the proportions of IPT3 uptake reported in this study (71%) and the national figure (39%), only underscore the different variations of IPTp uptake in Ghana. For instance, a similar study by Antwi in Bosomtwi district found that over 95% of received at least one dose of SP, which is consistent with the findings of this study [19]. The educational level of the study participants was significantly associated with the optimal (≥3 doses of SP) uptake of IPTp-SP. This might be due to the fact that education enables people to be well informed and make informed decisions and choices especially on maternal health issues. Women who are educated better appreciate the complications associated with pregnancy and childbirth and are more likely to receive optimal doses of SP [20]. Also, Sunyani Township is both a municipality and a regional capital, thus majority of the pregnant women were probably in the working group, therefore better educated and informed. It is expected that women who have given birth before (multiparous) would have had knowledge of the effects of malaria in pregnancy and IPTp because they would have probably been educated from their previous pregnancies. On the contrary, this study found no significant association between parity and uptake of IPTp-SP. Similar findings by Amoran et al in Western Nigeria and Takem et al in Cameroon found that parity had no significant effect on receiving optimal doses of SP [21, 22]. This could possibly be because women are likely to forget information that was given to them during previous pregnancies or could have suffered side effects of SP in previous pregnancies and will therefore not want to take it in subsequent pregnancies. In contrast to these findings, Antwi in Bosomtwe district found that women with history of previous deliveries were more likely to comply with the optimal uptake of SP during pregnancy [19].

More than 70% of respondents in this study had good knowledge of IPTp which was a significant determinant of uptake of optimal doses of SP in the Sunyani Municipality. This study result is not different from results of other studies which reported that knowledge of IPTp is significantly related to uptake of IPTp [19, 21, 23]. It is therefore not surprising that studies that reported low level of knowledge of IPT among pregnant women resulted in low uptake of IPTp [24]. This probably explains why health education among pregnant women on IPTp will better improve their knowledge level and increase uptake. The study also reported majority of the respondents of having good knowledge of the cause of malaria in pregnancy. Over 80% of them attributed the cause of malaria to be as a result of mosquito bites. This was consistent with findings revealed by Akaba et al in Nigeria [25]. However, the knowledge of the women on effects of malaria in pregnancy was low; this observation is in line with findings by Enato et al [26]. This low knowledge of effects of MiP have serious consequences on both the mother and the unborn child. Meanwhile, the association between perceived knowledge of the effects of MiP and uptake of IPTp was not significant in previous studies [27]. Early first ANC is essential to receiving optimal doses of SP. This is because a woman making early first ANC visit is more likely to receive more doses of SP if backed with frequent ANC visits and is provided with the SP at the facility. This study found that majority of the women made their first ANC visit at a median gestational age of 15 weeks which is comparable to findings in Bosomtwe district where the median gestational age at first ANC was 16 weeks [19]. This implies that women in the Sunyani Municipality attend antenatal clinics at an early gestational age but this did not make a significant difference on SP uptake. This is similar to findings by a study in Northern Nigeria that found that early gestation at booking was not associated with IPTp uptake among pregnant women [21]. Over 90% of the respondents in this study made four or more ANC visits. The number of recommended visits by WHO is at least four visits for no risk pregnancy. This study observed that attending antenatal care for four or more times was significantly associated with receiving optimal doses of SP. This finding is in consonance with many other studies that demonstrated that making four or more ANC visits is significantly related to receiving more doses of SP [19, 28, 29]. The age group and marital status of respondents was not significantly associated with the uptake of optimal doses of SP among pregnant women. This finding was inconsistent with studies conducted in Tanzania that observed that age group and marital status of pregnant women influenced the uptake of optimal doses of SP [30].

## Conclusion

This study has demonstrated that education, having a good knowledge of IPTp and malaria in pregnancy as well as four or more ANC visits significantly influenced the uptake of optimal IPTp doses. Education of women on IPTp and malaria in pregnancy should be intensified. Follow up visits or contacts should also be made on pregnant women who initiate ANC to encourage them to return for SP.

### What is known about this topic

- Maternal education influences the uptake of optimal doses of SP;
- Having four or more ANC visits is positively associated with optimal uptake of SP;
- Knowledge of malaria in pregnancy influences the uptake of optimal doses of SP.

### What this study adds

- Knowledge of IPTp in the Sunyani Municipality influences the uptake of optimal doses of SP;
- This study has shown that early initiation of ANC does not necessarily influence the uptake of optimal doses of SP as reported in other studies.

## Competing interests

The authors declare no competing interests.
